# Efficient Biocatalytic Preparation of Rebaudioside KA: Highly Selective Glycosylation Coupled with UDPG Regeneration

**DOI:** 10.1038/s41598-020-63379-9

**Published:** 2020-04-10

**Authors:** Yunyun Zhang, Shaohua Xu, Yue Jin, Yan Dai, Yijun Chen, Xuri Wu

**Affiliations:** State Key Laboratory of Natural Medicines and Laboratory of Chemical Biology, China Pharmaceutical University, Nanjing, 211198 China

**Keywords:** Biocatalysis, Enzymes, Glycobiology

## Abstract

Rebaudioside KA is a diterpene natural sweetener isolated in a trace amount from the leaves of *Stevia rebaudiana*. Selective glycosylation of rubusoside, a natural product abundantly presented in various plants, is a feasible approach for the biosynthesis of rebaudioside KA. In this study, bacterial glycosyltransferase OleD was identified to selectively transfer glucose from UDPG to 2′-hydroxyl group with a *β*-1,2 linkage at 19-COO-*β*-D-glucosyl moiety of rubusoside for the biosynthesis of rebaudioside KA. To eliminate the use of UDPG and improve the productivity, a UDPG regeneration system was constructed as an engineered *Escherichia coli* strain to couple with the glycosyltransferase. Finally, rubusoside at 22.5 g/L (35.0 mM) was completely converted to rebaudioside KA by the whole cells without exogenous addition of UDPG. This study provides an efficient and scalable method for highly selective biosynthesis of rebaudioside KA.

## Introduction

**Figure 1:**
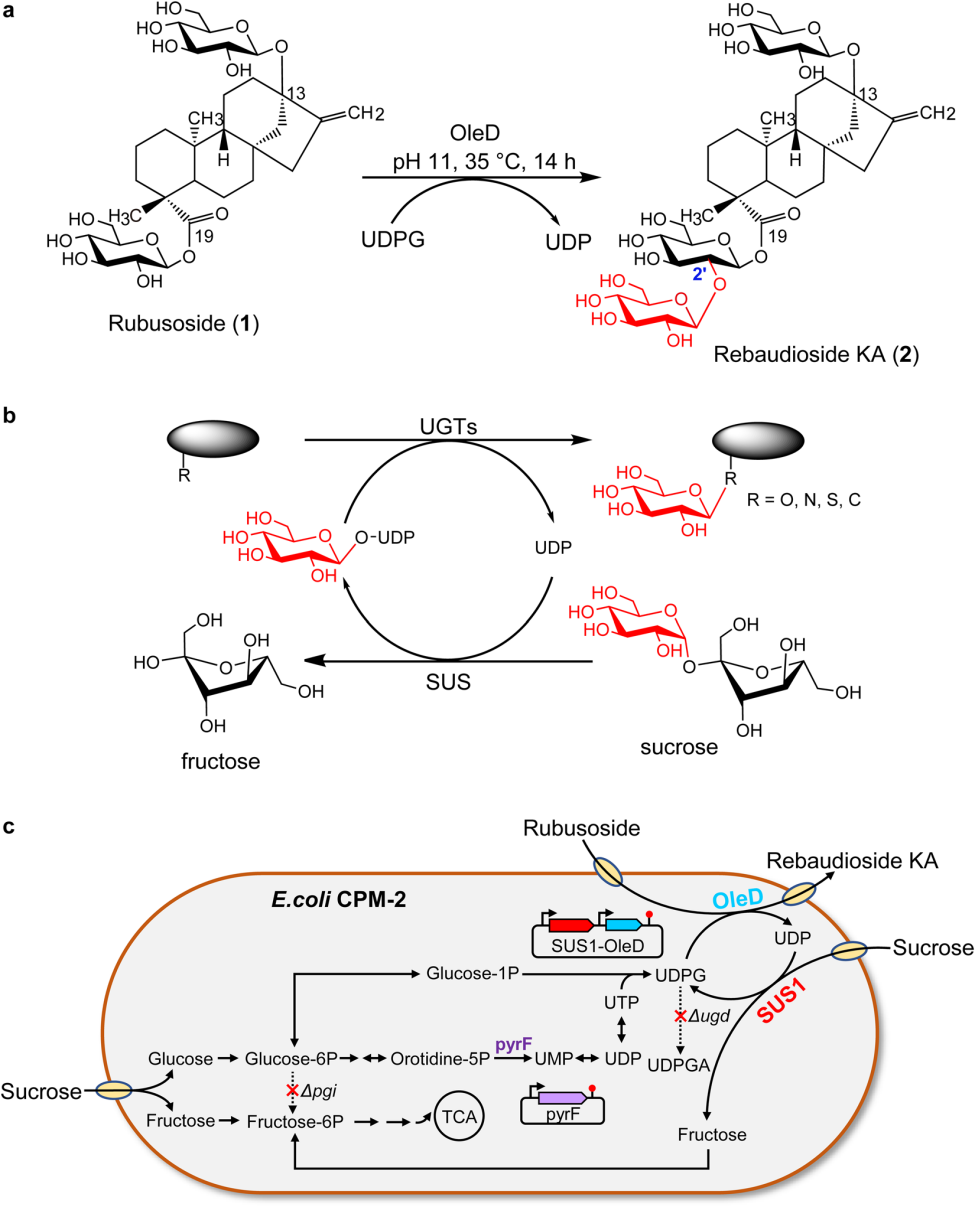
Biosynthesis of rebaudioside KA from rubusoside. (**a**) Scheme of the glycosylation of rubusoside by OleD. (**b**) Cascade reaction to couple the glycosylation of target compound (black ellipse) with UDPG regeneration. (**c**) Schematic diagram for improvement of rebaudioside KA production in *E.coli*. Abbreviation: OleD, glycosyltransferase; SUS1, sucrose synthase; pyrF, orotidine-5′-phosphate decarboxylase; pgi, glucose-6-phosphate isomerase; ugd, UDPG dehydrogenase; UDPGA, uridine diphosphate glucuronic acid, TCA, tricarboxylic acid.

Rubusoside (*β*-D-glucosyl ester of 13-O-*β*-D-glucosyl-steviol) (**1**, Fig. 1a) is a safe, stable and low-calorie sweetener, possessing 114-fold more sweetness than sucrose at a concentration of 0.025%^1^. It was originally isolated from the leaves of *Rubus chingii* Hu and *Rubus suavissimus* S. Lee in a high yield (5.3%)^2,3^. In 2016, rubusoside and various related glycosides were designated as “Generally Recognized as Safe” (GRAS) by the U.S. Food and Drug Administration (FDA). Additionally, it shows a variety of biological activities, including anti-hypertensive, anti-hyperglycaemic, anti-bacterial and anti-inflammation^4,5^. However, rubusoside exhibits a bitter aftertaste, which greatly undermines its commercial value. To improve the sweetness and the taste of rubusoside, tremendous efforts have been made to search for chemically and enzymatically modified derivatives during the past several decades^6–10^. Meanwhile, various sugar moieties, such as glucosyl, fructosyl and galactosyl, have been introduced at different hydroxyl groups of rubusoside. Unfortunately, the outcomes have not been satisfactory thus far.

Rebaudioside KA (**2**, Fig. 1a), a mono-glycosylated derivative of rubusoside, was isolated in a trace amount from the leaves of *Stevia rebaudiana*^11^, which sheds a new light on the discovery of novel natural sweeteners. Rebaudioside KA shows higher sweetness and more pleasant tasting than rubusoside. In particular, rebaudioside KA does not exhibit bitter aftertaste tested by 35 healthy volunteers (unpublished data), which makes it as an ideal candidate for natural sweeteners. Since trace amount of rebaudioside KA has been found only in one plant, the possibility of isolating this compound from natural sources is extremely low. Meanwhile, given the abundance in nature, the commercial availability and the same diterpene skeleton, stereo- and regio-specific glycosylation at 2′-hydroxyl group of C_19_-COO-*β*-D-glucosyl moiety of rubusoside could produce rebaudioside KA in a large quantity. Therefore, the exploration of selective glycosylation of rubusoside is a feasible way for the generation of rebaudioside KA.

To date, a variety of enzymes have been used for the glycosylation of rubusoside, including cyclodextrin glycosyltransferase^7^, *β*-galactosidases^8^, *β*-fructofuranosidase^9^ and dextransucrase^10^. Although these enzymes have shown the potential on the glycosylation of rubusoside, low productivity (2.7–66%) and the co-existence of multiple products have been major issues for their usefulness. By contrast, based on the versatility and the flexible binding pockets, UDP-glycosyltransferases (UGTs) could be a class of valuable enzymes to achieve the selective glycosylation of rubusoside to produce rebaudioside KA.

Although UGTs have been used to glycosylate various natural products, such as flavonoids, phenylpropanoids and terpenoids, the absolute requirement of costly sugar donor UDP-glucose (UDPG) has markedly limited their applicability^12^. To overcome this limitation, UDPG regeneration system has been subsequently developed^13–16^, in which UGT is coupled with a sucrose synthase to recycle UDPG with inexpensive sucrose in a cascade reaction (Fig. 1b). Due to the lower intracellular concentration of UDPG (usually 1–2 mM)^17^, direct regeneration of UDPG in the organisms has been difficult to accomplish. Consequently, various approaches on the engineering of microbial hosts to increase intrinsic UDPG and provide a continuous supply have been explored through the enhancement of natural metabolic pathways^18–20^ or the introduction of heterologous ones^21,22^. However, these engineered strains have typically resulted in incomplete conversion or low productivity (substrate concentration usually less than 10 mM) for the glycosylation, because current focus on UDPG biosynthesis has mainly dealt with glycolysis, pentose phosphate pathway and glycogen formation^18,20^. In fact, UTP and UDP are the precursors of UDPG in pyrimidine biosynthetic pathway^17^, and their availability may also be vital for UDPG biosynthesis. Thus, the efficiency of UDPG regeneration and simultaneous glycosylation could be improved from the increase of UTP and UDP.

In this study, a glycosyltransferase OleD from *Streptomyces antibioticus*, possessing the capability of glycosylating diverse types of scaffolds to form O-, N- and S-linked glucosides^23^, was identified to selectively catalyze the conversion of rubusoside to rebaudioside KA. Moreover, a self-sufficient system for UDPG was constructed and optimized in engineered *E. coli* by coupling OleD with a sucrose synthase (SUS1) to efficiently produce rebaudioside KA without exogenous addition of expensive UDPG (Fig. 1c). The present study offers an opportunity of preparing rebaudioside KA.

## Results and Discussion

### Enzyme screening and confirmation of the glycosylation of rubusoside

According to previous reports, five UGTs with broad aglycon tolerance, large binding pocket, and potential *β*-1,2 selectivity were selected for the preparation of *β*-1,2-mono-glycosylated rubusoside (Supplementary Table S1). These enzymes included YjiC from *Bacillus licheniformis*^24^, OleD from *Streptomyces antibioticus*^25^, GtfE from *Amycolatopsis orientalis*^26^, GtfB from *Amycolatopsis orientalis* and a chimeric glycosyltransferase GtfAH1^27^. After gene cloning and protein expression (Supplementary Fig. S1), the crude extract containing the glycosylation product was analyzed by HPLC and LC-MS (Supplementary Fig. S2). Among the five enzymes, just OleD and YjiC were able to glycosylate rubusoside. From mass spectrometry analysis, compound **2** ([M − H]^−^ *m/z*^−^ ~803.37), **3**, **4**, **5** ([M + Na]^+^ *m/z*^+^ ~827.37) were found to be mono-glycosylated derivatives of rubusoside (C_32_H_50_O_13_, molecular weight ~642.74) respectively. The glycosyltransferase OleD could completely convert rubusoside to compound **2** as a single product, which was quite unusual to any types of glycosylation. Although YjiC was able to simultaneously produce mono-glycosylated compounds **3**, **4** and **5**, the nature of multiple products severely limited its usefulness. Therefore, compounds **3**, **4** and **5** were not isolated for structural determination.

After purification by semi-preparative HPLC, 142 mg of compound **2** were obtained from the reaction mixture and its structure was elucidated by 1D- and 2D-NMR spectroscopy (Supplementary Figs. S3–S9). Compared to reported NMR spectra of rubusoside^28^, the downfield ^13^C shift (~4.26 ppm) at C-2′ of C_19_-COO-*β*-D-glucosyl moiety and the couple constant (*J* = 7.8 Hz) of the corresponding anomeric proton indicated that a glucosyl moiety was specifically added to 2′-hydroxyl group of C_19_-*O*-glucose of rubusoside in a *β*-configuration. ^1^H and ^13^C NMR spectra of compound **2** (Supplementary Table S2) were in accordance with the data previously reported, thus confirming compound **2** to be rebaudioside KA^11^. More importantly, given the generation of a single glycosylated product, OleD exhibited exceptional stereo- and regio-selectivity towards rubusoside. In addition, its excellent catalytic efficiency made it to be an ideal enzyme for the glycosylation of rubusoside.

### Optimization of glycosylation conditions

**Figure 2:**
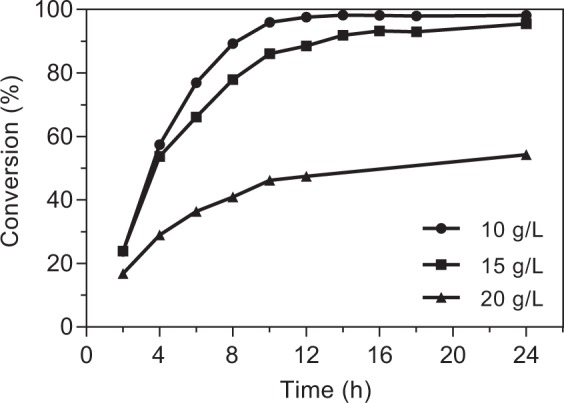
Time course for the biosynthesis of rebaudioside KA with crude extract containing OleD under optimized conditions.

To simplify the enzymatic synthesis and improve the yield of rebaudioside KA, a reaction system using crude enzyme extract without extensive protein purification was established. The OleD-catalyzed conversion of rubusoside under different pH (6.0–12.0), temperature (15–40 °C), rubusoside concentration (7.5–25 g/L), molar ratio of rubusoside/UDPG (1:1–1:8) and reaction time (2–24 h) were compared to optimize the glycosylation conditions (Supplementary Fig. S10 and Fig. 2). The highest conversion of rubusoside was achieved in 50 mM sodium phosphate (pH 11.0) buffer at 35 °C. The rubusoside/UDPG molar ratio was found to be optimal at 1:6. When the concentration of rubusoside increased from 7.5 g/L to 25 g/L, the conversion decreased from 98.0% to 88.5%, and then rapidly declined to 29.5% (Supplementary Fig. S10). Subsequently, the time course for rebaudioside KA production from rubusoside was monitored at different substrate concentrations (10, 15 and 20 g/L) under the optimized conditions. As shown in Fig. 2, rebaudioside KA could be efficiently biosynthesized during the first 10 h. After 24 h of reaction, 10 g/L and 15 g/L rubusoside were completely glycosylated (>95%), whereas 54.3% of rubusoside was converted at concentration of 20 g/L. At high substrate concentrations, the accumulation of UDP during the reaction could be an important factor for the suppression of the catalytic efficiency and the promotion of reverse glycosylation^29^. Although OleD could completely convert 15 g/L rubusoside to rebaudioside KA, the addition of costly UDPG markedly prohibited its practical application.

### Construction of the *E. coli* strain for the regeneration of UDPG

**Figure 3:**
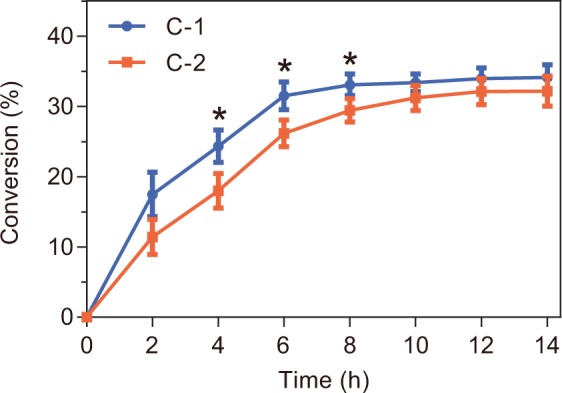
Catalytic efficiency of *E. coli* C-1 and C-2 for the biosynthesis of rebaudioside KA. (*) indicates a significant difference in the conversion between *E. coli* strains of C-1 and C-2.

To reduce the use of expensive UDPG, a UDPG regeneration system coupling with the glycosylation by OleD was constructed in *E. coli* cells. Given the poor stability and the difficulty on the quantification of UDPG in the dynamic system, the regeneration efficiency was evaluated based on the production of rebaudioside KA from rubusoside by *E. coli* cells. Firstly, the genes of OleD and a widely used sucrose synthase (SUS1) from *Arabidopsis thaliana* were synthesized and constructed in pETDuet-1 vector preceded by a T7 promoter/*lac* operon. To compare the effects of cloning order on regeneration efficiency, two genes were inserted into different multiple cloning sites to yield recombinant plasmids pAT-GT and pGT-AT respectively. These two constructs were expressed in *E. coli* BL21(DE3), and the corresponding strains *E. coli* C-1 and C-2 (Supplementary Fig. S11a) were incubated in a mixture of 2 g/L rubusoside and 600 g/L sucrose, with the supply of UDPG (molar ratio of rubusoside/UDPG = 1:0.5) for the initiation of the UDPG recycling. *E. coli* C-1 showed higher conversion than C-2 (Fig. 3). Thus, C-1 strain was selected for subsequent experiments.

**Figure 4:**
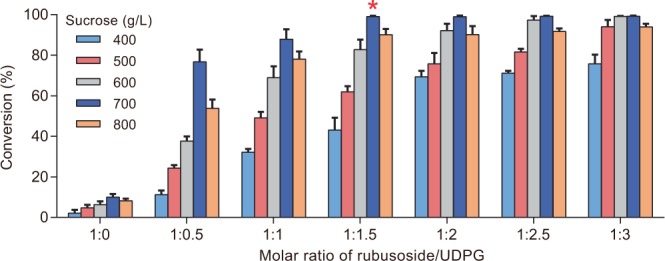
The efficiency of the UDPG regeneration system at different concentrations of sucrose and UDPG. (*) represents the optimal combination.

To examine whether the amount of UDPG could be reduced, the efficiency of the whole cell system was compared with different amounts of UDPG and sucrose. As shown in Fig. 4, when the concentration of sucrose reached 700 g/L and the molar ratio of rubusoside/UDPG = 1:1.5, 2 g/L rubusoside could be completely converted to rebaudioside KA, decreasing the demand for UDPG by 75%. The requirement of high concentrations of sucrose might be due to its high *K*_m_ value for sucrose synthase when expressed in prokaryotic hosts^30^. Since the activities of OleD and sucrose synthase are strongly pH-dependent but they present distinct optimal pH values^31^, a lower conversion as shown in Fig. 4 was not surprising. In addition, the conversion of 2 g/L rubusoside in the OleD-SUS1 cascade system was only 10.0% in the absence of UDPG, suggesting that the introduction of sucrose synthase was not enough to drive UDPG regeneration. Thus, further engineering of the host and optimization of whole-cell reaction is required.

### Improvement of metabolic flux toward UDPG synthesis

**Figure 5:**
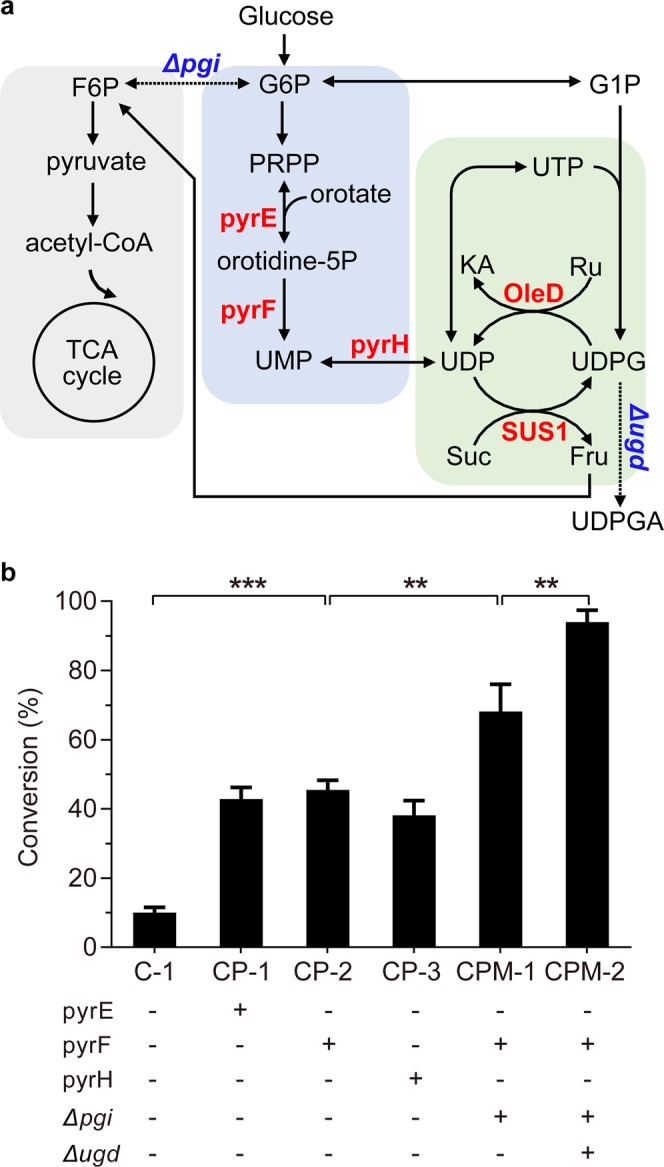
Construction of redirected carbon metabolic pathways for UDPG biosynthesis. (**a**) Engineering strategies for broadening the metabolic flux toward UDPG biosynthesis in *E. coli* C-1. Overexpressed genes are shown in red and deleted genes are shown in blue with italic, (*Δ*) represents gene knock-out. The glycolysis, pyrimidine metabolism and regeneration cycle are expressed by gray, blue, and green rounded rectangles, respectively. Abbreviations: F6P, fructose-6-phosphate; TCA, tricarboxylic acid; G6P, glucose-6-phosphate PRPP, 5-phosphoribosyl-1-pyrophosphate; UMP, uridine monophosphate; UDP, uridine diphosphate; UTP, uridine triphosphate; UDPG, uridine diphosphate glucose; UDPGA, uridine diphosphate glucuronic acid; KA, rebaudioside KA; Ru, rubusoside; Suc, sucrose; Fru, fructose; G1P, glucose-1-phosphate. (**b**) Comparison of rubusoside conversion by the engineered strains. **p < 0.01 and ***P < 0.001 indicate the statistical significances between different groups.

Given the low conversion with the heterologous expression of sucrose synthase and the supply of sucrose, we supposed that lower intrinsic amount of UDP may result in a low efficiency on UDPG regeneration and the simultaneous bioconversion. UDP is primarily synthesized from the pyrimidine metabolic pathway in *E. coli*^32^, in which orotate phosphoribosyltransferase (pyrE) and orotidine-5′-phosphate decarboxylase (pyrF) are involved in UMP biosynthesis from orotate, while uridylate kinase (pyrH) catalyzes the phosphorylation of UMP to UDP (Fig. 5a). Therefore, enhancement of the pyrimidine biosynthetic pathway through fine-tuning the metabolic flux should promote UDPG production. To test our hypothesis, these upstream enzymes were individually overexpressed in *E. coli* strain C-1 to yield recombinant strains of CP-1, CP-2 and CP-3 respectively (Supplementary Fig. S11b). Among these strains, CP-2 showed a better conversion, possibly due to the fact that pyrF catalyzes the irreversible reaction with UMP formation. After 12 h, CP-2 strain converted 2 g/L rubusoside to rebaudioside KA with a conversion of 45.5%, significantly higher than that of C-1 (Fig. 5b). However, when pyrE, pyrF and pyrH were co-expressed in C-1 strain, the conversion was unexpectedly lower than that of CP-2 (Supplementary Fig. S12), suggesting that the pyrimidine metabolic pathway is more complicated than current knowledge and requires further clarification.

Besides enhancing the UDP biosynthetic pathway, redistribution of carbon flux should be advantageous for the UDPG regeneration. As previously known, glucose-6-phosphate (G6P) is crucial in directing carbon source to glycolysis, pentose phosphate pathway, and the conversion to glucose-1-phosphate (G1P) (Fig. 5a). Knockout of G6P isomerase (*pgi*) could accumulate UDPG by increasing the G6P pool through the elimination of acetate formation and the excessive metabolism of *E. coli*^33,34^. Thus, compared to the reference strain CP-2, the conversion of rubusoside showed 50% higher in the *pgi*-deleted *E. coli* strain (CPM-1), in which the glycolysis pathway was blocked (Fig. 5b).

The decreasing of the metabolic consumption of UDPG represents another way to increase the availability of intracellular UDPG. Because UDP-glucose dehydrogenase (*ugd*) is a major enzyme for UDPG degradation^35^, an *E. coli* strain (CPM-2) was created by deleting *ugd* in CPM-1, resulting in the increasing of rubusoside conversion to 94.0% without UDPG addition (Fig. 5b). These results confirmed that an UDPG self-sufficient *E. coli* strain was successfully constructed. By introducing the glycosyltransferase OleD, rubusoside at 2 g/L was completely converted to rebaudioside KA without the addition of UDPG, which may reflect the collective contribution of the blockage of glycolysis, the increasing of UDP formation, and the prevention of UDPG degradation in *E. coli*.

### Maximization of UDPG self-sufficient system for rebaudioside KA production

The engineered *E. coli* strain (CPM-2) was then used to perform the bioconversion for rebaudioside KA. To maximize the efficiency for the obtainment of rebaudioside KA, the reaction parameters involved in the biosynthesis were further optimized, including temperature, pH, sucrose concentration, rubusoside concentration and bacterial biomass. Firstly, sucrose (700 g/L), rubusoside (12.5 g/L) and bacterial biomass (25 g/L) in a total volume of 1 mL were used for the evaluation of temperature (25–55 °C) and pH (5.5–10.0). The optimal conditions of 40 °C and pH 7.0 were obtained (Supplementary Fig. S13). Then, we designed a three-factor five-level orthogonal experiment to analyze the influence of sucrose, rubusoside and biomass on the production of rebaudioside KA (Supplementary Tables S3 and S4). The results of the orthogonal experiment were examined by variance analysis (Supplementary Table S5). Based on F and P values, the significant influencing factors were sucrose and bacterial biomass, and rubusoside had the least effect on the product yield. From the estimated marginal means of factors (Supplementary Table S6), the optimal combination for the highest production was 800 g/L sucrose, 22.5 g/L rubusoside and 100 g/L bacterial biomass, which was subsequently confirmed experimentally at a larger scale.

**Figure 6:**
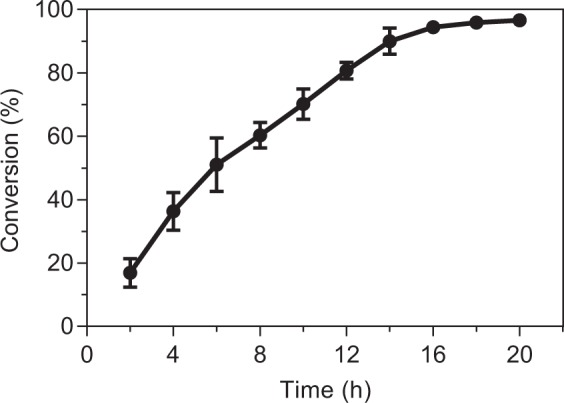
Time course for the biosynthesis of rebaudioside KA by engineered *E. coli* CPM-2 under optimized conditions.

The whole cell bioconversion was carried out in M9 medium (pH 7.0) containing 800 g/L sucrose, 22.5 g/L rubusoside and 100 g/L bacterial biomass in a 200 mL volume at 40 °C, 220 rpm for 20 h. During the glycosylating process, samples were taken every 2 h for HPLC analysis to calculate the conversion of rubusoside. After 18 h of the reaction, rubusoside was completely converted to rebaudioside KA (>95%) (Fig. 6), demonstrating the compatibility between the UDPG regeneration system and the selective glycosylation by glycosyltransferase OleD. Compared to other UDP-glycosyltransferases coupled with different UDPG regeneration approaches for the glycosylation of various natural products^12,31^, the present method has showed obvious advantages on substrate concentration and productivity.

## Conclusion

Glycosyltransferase OleD was identified to selectively glycosylate rubusoside for the generation of rebaudioside KA. An engineered *E. coli* strain of CPM-2 was constructed to regenerate UDPG. In addition to the blockage of glycolysis and the prevention of UDPG degradation, the increasing of the biosynthetic precursors of UDPG was confirmed to be important for the regeneration of UDPG in *E. coli* cells. By coupling glycosyltransferase OleD with the engineered *E. coli* strain, rebaudioside KA could be efficiently produced from rubusoside at 22.5 g/L (35.0 mM) substrate concentration without exogeneous addition of UDPG. The present glycosyltransferase-UDPG regeneration system can completely eliminate the use of UDPG and enable the selective and efficient biosynthesis of various food-related glycosides.

## Materials and methods

### Chemical and biological materials

Rubusoside (purity 92%) was purchased from Tongtian Biotechnology Co., Ltd. (Shanghai, China). UDPG (purity 98%) was purchased from Ruixin Biotech Co. (Suzhou, China). All strains and plasmids used in this study are listed in Supplementary Table S7. Plasmids were constructed according to the standard protocols^36^. All PCR primers used for gene cloning were synthesized by GENEWIZ Co. (Suzhou, China) and are listed in Supplementary Table S8. The nucleotide sequences of codon-optimized UGTs (YjiC, OleD, GtfE, GtfB, GtfAH1) and SUS1 were synthesized by GENEWIZ. Gene deletion strains of *E. coli* BL21(DE3) were obtained using λ-Red deletion method^37^.

### Protein expression and functional verification of selected glycosyltransferases

The plasmids pET-302 or pET-22b (+) containing glycosyltransferase genes were respectively transformed into *E. coli* BL21(DE3). The recombinant strains were grown in Luria-Bertani (LB) medium containing 100 μg/mL ampicillin at 37 °C. Protein expression was performed according to previous report^25^. Cells were harvested by centrifugation at 5, 000 × g for 10 min and resuspended in 50 mM Tris-HCl buffer (pH 8.0). The cells were lysed by sonication on ice and the pellets were removed by centrifugation. The crude extracts were analyzed by SDS-PAGE.

The glycosylation of rubusoside by the selected UGTs was verified using the crude extracts of the recombinant cells. Reaction mixtures containing 600 μL crude extract, 2 g/L rubusoside and 7.8 g/L UDPG were incubated at 30 °C, 220 rpm for 12 h. The reactions were terminated by adding an equal volume of methanol. After centrifugation at 10, 000 × g for 10 min, the reaction mixtures were analyzed by HPLC on Agilent 1260 Infinity with a YMC-C18 column (250 mm × 4.6 mm; 5 μm). Analysis was performed at 35 °C with UV detection at 210 nm by water (containing 0.1% formic acid, solvent A) and acetonitrile (containing 0.1% formic acid, solvent B) at a flow rate of 1.0 mL/min under following gradient: 10%-90% solvent B for 25 min. LC-MS was performed at negative or positive ion mode on Agilent LC1200/MS Q-TOF 6520.

### Preparation and structural elucidation of rebaudioside KA

To elucidate the structure of mono-glycosylated rubusoside, the reaction was performed at 200 mL with the same conditions described above. The reaction was stopped by adding twice volume of methanol and centrifuged at 10, 000 × g for 10 min. Methanol and a part of water were removed under reduced pressure at 42 °C. The residual supernatant was lyophilized for 48 h. Subsequently, the lyophilized powder was dissolved in water and purified by Waters Prep150 semi-preparative HPLC. The purified product was dissolved in DMSO-*d*_6_ at a final concentration of 20 mg/mL and the structure was elucidated by 1D- and 2D-NMR.

### Optimization of enzymatic reaction conditions

The standard reaction of OleD with rubusoside was performed in 1 mL crude extract reaction mixtures containing 50 mM sodium phosphate (pH 11.0), 15 g/L rubusoside, 58.6 g/L UDPG (molar ratio of rubusoside/UDPG = 1:4) and incubated at 30 °C for 12 h. For pH optimization, 50 mM sodium phosphate, 50 mM Tris-HCl, 50 mM glycine-NaOH were used for the range of pH from 6.0 to 12.0. The optimal temperature was examined ranging from 15 °C to 40 °C. The concentration of rubusoside was investigated ranging from 7.5–25 g/L. The optimal molar ratio of rubusoside/UDPG was compared ranging from 1:1 to 1:8. For the optimization of reaction time, reaction was performed under the optimized conditions. The conversion (%) of rubusoside to rebaudioside KA was calculated as following: conversion (%) = C_KA_/C_Ru_, where C_KA_ represents the rebaudioside KA molar concentration during the reaction, C_Ru_ represents the initial rubusodide molar concentration.

### Whole-cell bioconversion

The engineered *E. coli* strains (C-1, C-2, CP-1, CP-2, CP-3, CPM-1, CPM-2) were respectively grown in LB medium containing appropriate antibiotics at 37 °C until OD_600_ = 0.8–1.0. IPTG was added at a final concentration of 1 mM, and protein expression was induced at 37 °C for 10 h. The cells were harvested by centrifugation at 5, 000 × g for 10 min. The pellet was resuspended in a modified M9 medium (pH 6.5) containing 12.8 g/L Na_2_HPO_4_·7H_2_O, 3 g/L KH_2_PO_4_, 0.5 g/L NaCl, 1 g/L NH_4_Cl and 2 mM MgCl_2_. Whole cells with UDPG and sucrose at different concentrations and 2 g/L rubusoside in a total volume of 1 mL (bacterial biomass 25 g/L) were incubated at 35 °C, 220 rpm for 12 h. All reactions were stopped by adding twice volume of methanol, and the products were analyzed by HPLC. All data are represented as the means of three independent experiments with standard deviations; Student T-test was applied for the comparison of mean values between two groups (*p < 0.05, **p < 0.01 and ***P < 0.001 indicate the statistical significances between different groups).

### Orthogonal experiment

To determine the optimal combination for rubusoside conversion in whole-cell reactions, concentrations of sucrose (500, 600, 700, 800, 900 g/L), rubusoside (15, 17.5, 20, 22.5, 25 g/L), and bacterial biomass (25, 50, 75, 100, 125 g/L) were tested though the orthogonal experiment. Firstly, 25 combinations were obtained using orthogonal array design L_25_ (5^3^). The different combination was mixed in 1 mL M9 medium (pH 7.0) and performed at 40 °C, 220 rpm for 12 h. The reactions were terminated with methanol and subjected to HPLC analysis. Subsequently, the production of rebaudioside KA as dependent variable was analyzed by variance analysis, which was conducted using IBM SPSS statistics 22.0 software based on the main effect model.

## Supplementary information

Supplementary information.
