# Supplementary material for: Efficient Biocatalytic Preparation of Rebaudioside KA: Highly Selective Glycosylation Coupled with UDPG Regeneration
Source: Sci Rep. 2020 Apr 10;10:6230. doi: 10.1038/s41598-020-63379-9 (PMC7148340; doi:10.1038/s41598-020-63379-9)
Supplement: Supplementary file 1 — Supplementary information. [file 41598_2020_63379_MOESM1_ESM.pdf]

Supplementary Information

Efficient Biocatalytic Preparation of Rebaudioside KA: Highly Selective Glycosylation Coupled with UDPG Regeneration

Yunyun Zhang[1,+], Shaohua Xu[1,+], Yue Jin[1], Yan Dai[1], Yijun Chen[1,*], and Xuri Wu[1,*]

[1]State Key Laboratory of Natural Medicines and Laboratory of Chemical Biology, China Pharmaceutical University, Nanjing, 211198, China

*Corresponding author. yjchen@cpu.edu.cn (Yijun Chen), xuriwu@cpu.edu.cn (Xuri Wu)

[+]These authors contributed equally to this work.

Supplementary Methods

Gene deletion

The primers associated with the gene deletions are listed in Table S1. Homology fragments for deletions of *pgi* and *ugd* genes were amplified using pKD3 as template and pgi-homo-F/R and ugd-homo-F/R as primers respectively. pKD46 for the expression of λ-Red recombinases was used for the recombination. The resistance gene was eliminated by a helper plasmid pCP20 encoding FLP recombinase. All mutants were verified by PCR with pgi-con-F/R and ugd-con-F/R as primers respectively and further confirmed by DNA sequencing.

Supplementary Data

Table S1. Summary of functionally characterized UDP-glycosyltransferases

Enzyme	GenBank No.	Substrate	Linkage	Reference
YjiC	AAU40842	flavonoids, isoflavonoids, chalcones, stilbenes	*β*-1, 2-<br>O-*β*-2-	1
OleD	ABA42119	oleandomycin flavones, isoflavones, alkaloids, stilbenes, steroids chalcones	*β*-1, 2-<br>O-*β*-2-	2,3
GtfE	AAK31353	vancomycin, teicoplanin,	O-*β*-2-	4
GtfB	AAB49293	vancomycin,	O-*β*-2-	4
GtfAH1	P96558	hybrid glycopeptides	O-*β*-2-<br>*β*-1, 2-	5

Table S2. ^{1}H NMR and ^{13}C NMR spectral assignments for compound **2** (in DMSO-d_6, J in Hz). [a]The solvent was C_5D_5N

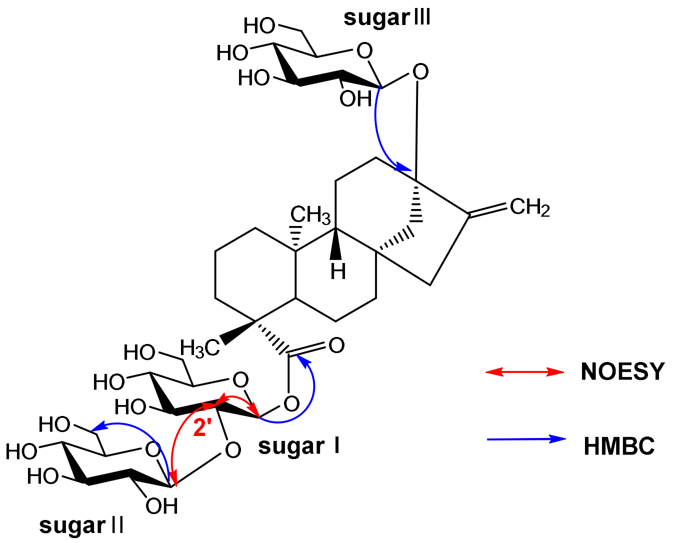

Sugar	Position	Compound **2**			Rebaudioside KA[a]	
		δC	δH（J in Hz）	HMBC	δC	δH
	1	39.95	1.82 (m)<br>0.79 (m)		40.8	1.75 (m)<br>0.76 (m)
	2	19.06	1.78 (m)<br>1.39 (m)		20.2	2.17 (m)<br>1.70 (m)
	3	37.51	1.87 (m)<br>1.42 (m)		38.9	2.14 (m)<br>1.82 (m)
	4	43.34			44.5	
	5	56.45	1.04 (dd,6.5,7.5)		57.6	0.99 (m)
	6	21.08	1.83 (m)		22.2	2.20 (m)<br>1.91 (m)
	7	40.94	1.53 (m)<br>1.39 (m)		41.9	1.51 (m)<br>1.31 (m)
	8	41.22			42.2	
	9	53.03	0.97 (br.d, 8.5)		54.2	0.93 (m)
	10	38.83			39.8	
	11	19.73	1.74 (m)<br>1.56 (m)		20.7	1.48 (m)
	12	36.55	2.34 (br.d, 12.8)<br>0.97 (br.d, 8.5)		38.0	2.75 (m)<br>1.10 (m)
	13	85.42			87.2	
	14	43.29	2.04 (s)		44.8	2.75 (m)

			1.51 (s)			1.94 (m)
	15	47.45	2.07 (m)		48.6	2.10 (m)
	16	152.36			153.8	
	17	104.39	5.17 (br. s)<br>4.80 (br. s)		105.5	5.10 (m)<br>5.64 (s)
	18	28.39	1.19 (s)		29.4	1.42 (s)
	19	174.55			176.1	
	20	15.95	0.85 (s)		16.5	0.99 (s)
I	1′	91.79	5.47 (d, 7.8)	78.26(C-2′);	97.7	5.12 (m)
	2′	78.26	3.57 (m)			
	3′	76.46	3.51 (m)			
	4′	70.17	3.05 (m)			
	5′	76.99	3.12 (m)			
	6′	61.15	3.47 (m)<br>3.71 (m)			
II	1″	103.38	4.54 (d, 7.7)	78.26 (C-2′);<br>77.51 (C-5″);	105.8	5.10 (m)
	2″	74.41	2.98 (m)			
	3″	76.52	3.17 (m)			
	4″	69.41	3.25 (m)			
	5″	77.51	3.27 (m)			
	6″	60.60	3.52 (m)<br>3.63 (m)			
III	1‴	97.79	4.32 (d, 7.7)	85.42 (C-13)	95.5	6.23 (bs)
	2‴	73.67	2.94 (m)			
	3‴	76.33	3.17 (m)			
	4‴	70.13	3.05 (m)			
	5‴	76.78	3.02 (m)			
	6‴	61.09	3.57 (m)<br>3.44 (m)			

Table S3. Factors and their levels employed in the orthogonal experiment

Levels	Factors		
	sucrose (g/L)	rubusoside (g/L)	biomass (g/L)
1	500	15	25
2	600	17.5	50
3	700	20	75
4	800	22.5	100
5	900	25	125

Table S4. Design of orthogonal experiment using L_{25} (5^3) array and the corresponding production of rebaudioside KA by engineered *E. coli* CPM-2 strain

No.	sucrose (g/L)	rubusoside (g/L)	biomass (g/L)	conversion (%)	production (g/L)
1	500	20	75	22.79	5.71
2	500	17.5	50	12.39	2.72
3	600	20	25	7.53	1.89
4	700	15	50	97.28	18.27
5	800	20	50	86.98	21.79
6	700	25	25	12.71	3.98
7	700	20	100	97.07	24.31
8	800	22.5	75	92.88	26.17
9	900	22.5	25	32.94	9.28
10	600	22.5	50	26.34	7.42
11	700	22.5	125	97.35	27.43
12	800	15	125	96.60	18.15
13	900	17.5	100	93.98	20.60

14	900	20	125	91.01	22.79
15	600	25	75	37.22	11.65
16	900	25	50	67.19	21.04
17	500	22.5	100	31.41	8.85
18	500	25	125	33.79	10.58
19	500	15	25	6.40	1.20
20	800	25	100	93.55	29.29
21	900	15	75	91.76	17.24
22	600	17.5	125	97.55	21.38
23	700	17.5	75	97.80	21.43
24	800	17.5	25	39.28	8.61
25	600	15	100	98.66	18.53

Table S5. Statistical tests between-subject effects (dependent variable: production). [a]R Squared = 0.869 (Adjusted R Squared = 0.737)

Source	Type III sum of squares	df	Mean Square	F value	P value
Corrected Model	1549.895[a]	12	129.158	6.607	0.001
Intercept	5785.332	1	5785.332	295.934	0.000
Sucrose	763.543	4	190.886	9.764	0.001
Rubusoside	3.773	4	0.943	0.048	0.995
Biomass	782.579	4	195.645	10.008	0.001
Error	234.593	12	19.549		
Total	7569.821	25			
Corrected Total	1784.488	24			

Table S6. Estimated marginal means of the factors in the orthogonal experiment (dependent variable: production).

Factor	Level[a]	Mean	95% Confidence Interval	
			Lower Bound	Upper Bound
Sucrose (g/L)	500	5.811	1.503	10.119
	600	12.174	7.866	16.483
	700	19.086	14.778	23.394
	800	20.801	16.492	25.109
	900	18.189	13.881	22.497
Rubusoside (g/L)	15	14.678	10.370	18.987
	17.5	14.947	10.638	19.255
	20	15.298	10.990	19.606
	22.5	15.831	11.523	20.139
	25	15.308	10.999	19.616
Biomass (g/L)	25	4.922	0.683	9.300
	50	14.247	9.939	18.555
	75	16.440	12.132	20.748
	100	20.316	16.008	24.625
	125	20.066	15.758	24.375

[a]Optimal combination is highlighted with yellow background.

Table S7. Plasmids and strains used in this study

Plasmid/Strain	Characteristic[a]	Reference
Plasmid		
pET-302	P_{T7}, pBR322 ori, Amp^r	Novagen
pET-22b (+)	P_{T7}, pBR322 ori, Amp^r	Novagen
pETDuet-1	P_{T7}, pBR322 ori, Amp^r	Novagen
pACYCDuet-1	P_{T7}, P15A ori, Cm^r	Novagen
pCDFDuet-1	P_{T7}, CloDF13 ori, Str^r	Novagen
p302-YjiC	pET-302 containing *YjiC*	this study
p22b-OleD	pET-22b (+) containing *OleD*	this study
p22b-GtfE	pET-22b (+) containing *GtfE*	this study
p22b-GtfB	pET-22b (+) containing *GtfB*	this study
p22b-GtfAH1	pET-22b (+) containing chimeric gene *GtfAH1*	this study
pAT-1	pETDuet-1 containing *SUS1* in MSC I	this study
pAT-2	pETDuet-1 containing *SUS1* in MSC II	this study
pAT-GT	pETDuet-1 containing *SUS1*and *OleD* cloned in MCS I and MCS II respectively	this study
pGT-AT	pETDuet-1 containing *OleD* and *SUS1* cloned in MCS I and MCS II respectively	this study
pCD-E	pCDFDuet-1 containing *pyrE*	this study
pCD-F	pCDFDuet-1 containing *pyrF*	this study
pCD-H	pACYCDuet-1 containing *pyrH*	this study
pKD46	expression plasmid of recombinases under ParaBAD promotor, Amp^r	6
pKD3	template for amplification of homology fragments, Cm^r	6
pCP20	helper plasmid expressing FLP recombinase for	7

	elimination of chloramphenicol resistence genes, Amp^r, Cm^r	
Strain		
BL21(DE3)	*Escherichia coli* BL21(DE3)	TIANGEN
BL21-YjiC	BL21(DE3) carrying p302-YjiC plasmid	this study
BL21-OleD	BL21(DE3) carrying p22b-OleD plasmid	this study
BL21-GtfE	BL21(DE3) carrying p22b-GlfE plasmid	this study
BL21-GtfB	BL21(DE3) carrying p22b-GtfB plasmid	this study
BL21-GtfAH1	BL21(DE3) carrying p22b-GtfAH1 plasmid	this study
C-1	BL21(DE3) carrying pAT-GT plasmid	this study
C-2	BL21(DE3) carrying pGT-AT plasmid	this study
CP-1	BL21(DE3) carrying pAT-GT and pCD-pyrE plasmid	this study
CP-2	BL21(DE3) carrying pAT-GT and pCD-pyrF plasmid	this study
CP-3	BL21(DE3) carrying pAT-GT and pA-pyrH plasmid	this study
Δpgi	BL21(DE3) with deletion of gene *pgi*	this study
Δpgi-Δugd	*Δpgi* strain with deletion of gene *ugd*	this study
CPM-1	*Δpgi* strain carrying pAT-GT and pCD-pyrF plasmid	this study
CPM-2	*Δpgi-Δugd* strain carrying pAT-GT and pCD-pyrF plasmid	this study

[a]Amp, ampicillin; Str, streptomycin; Cm, chloramphenicol; and r, resistance, MCS, multiple cloning site.

Table S8. Primers used in this study

Primer	Sequence (5'-3')[a]
**Primers used for plasmid construction of pAT-GT and pGT-AT**	
AT-NH-F	CATGCCATGGGCCAATGCAGAGCGCATG
AT-NH-R	CCCAAGCTTTTAATGATGGTGATGGTGATGATCG
AT-NX-F	CGCCATATGGCCAATGCAGAGCGCATG
AT-NX-R	CCGCTCGAGTTAATGATGGTGATGGTGATGATCG
GT-NH-F	CATGCCATGGATACCACCCAGACCACTCC
GT-NH-R	CCCAAGCTTTCACCCACCGTTGGGTCG
GT-NX-F	CGCCATATGATACCACCCAGACCACTCC
GT-NX-R	CCGCTCGAGTCACCCACCGTTGGGTCG
**Primers used for plasmid construction of pCD-E, pCD-F and pA-H**	
E-NH-F	CATGCCATGGGCATGAAACCATATCAGCGCCAG
E-NH-R	CCCAAGCTTTTAAACGCCAAACTCTTCGC
F-NX-F	GGAATTCCATATGACGTTAACTGCTTCATCTTCTTC
F-NX-R	CCGCTCGAGTCATGCACTCCGCTGTAAAG
H-NX-F	GGAATTCCATATGGCTACCAATGCAAAACCCGTCT
H-NX-R	CCGCTCGAGTTATTCCGTGATTAAAGTCCCTTCT
**Primers used for amplification of the homology fragment for deletion of *pgi* and *ugd* genes**	
pgi-homo-F	CGCTACAATCTTCCAAAGTCACAATTCTCAAAATCAGAAG AGTATTGCTAGTGTAGGCTGGAGCTGCTTC
pgi-homo-R	GTTGCCGGATGCGGCGTGAACGCCTTATCCGGCCTACATA TCGACGATGAATGGGAATTAGCCATGGTCC
ugd-homo-F	CGCAAGTAACAAAAGACAATCAGGGCGTAAATAGCCCTG ATAACAGGATGGTGTAGGCTGGAGCTGCTTC
ugd-homo-R	GATGCTAAAAACATCATGATTCACAGTTAAGTTAATTCTG

	AGAGCATGAAATGGGAATTAGCCATGGTCC
**Primers used for verification of the deletion of *pgi* and *ugd* genes**	
pgi-con-F	CAACATTACGCTAACGGCACT
pgi-con-R	CTGTAGGCCTGATAAGACGC
ugd-con-F	CAAAAGACAATCAGGGCGTAAATAG
ugd-con-R	TCACAGTTAAGTTAATTCTGAGAGC

[a]Restriction sites are underlined.

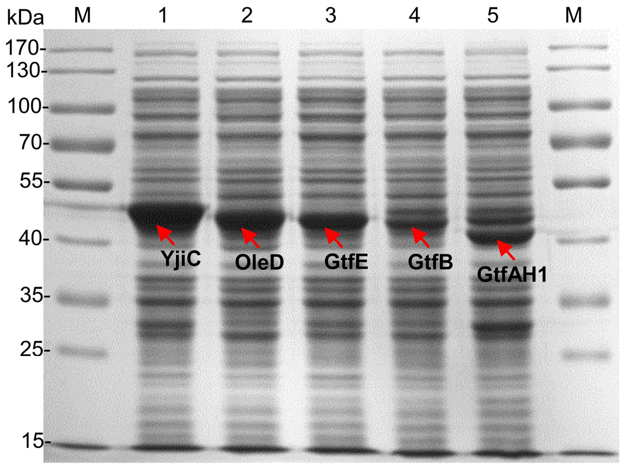

Figure S1. SDS-PAGE of the UDP-glycotransferases overexpressed in *E. coli* BL21. Molecular weight of target protein: YjiC, 44.6 kDa; OleD, 45.3 kDa; GtfE, 43.1 kDa; GtfB, 42.7 kDa; GtfAH1, 41.6 kDa.

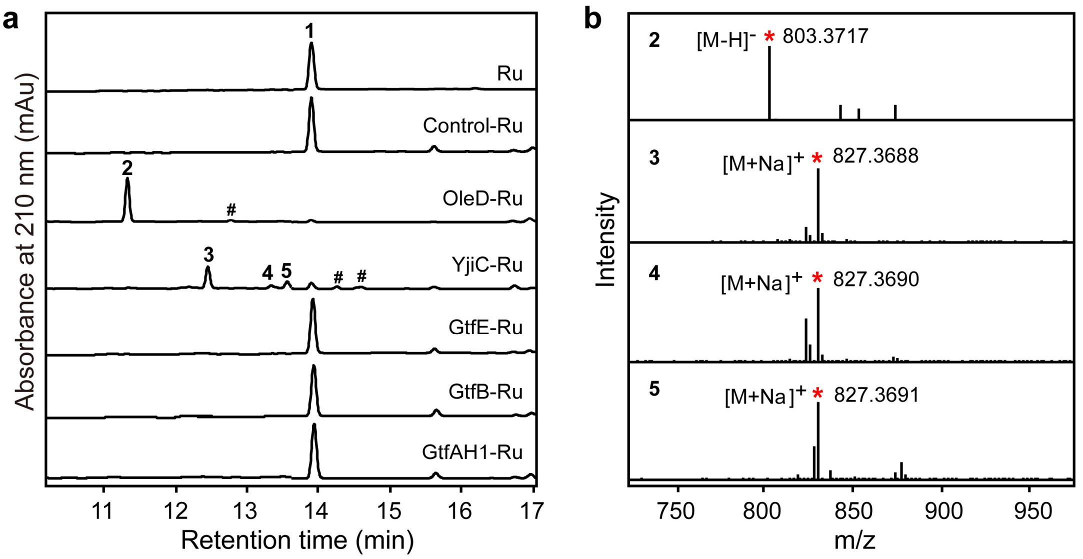

Figure S2. HPLC and MS analysis of glycosylated products of rubusoside. (a) HPLC chromatograms of the glycosylated products of rubusoside. (#) indicates unrelated impurities based on LC-MS analysis. Abbreviations: Ru, rubusoside. Control-Ru, the reaction was carried out with the crude extract of *E. coli* BL21(DE3)-pET22b, rubusoside and UDPG. (b) LC-MS results of the glycosylated products of rubusoside. (*) indicates the molecular ion of glycosylated products.

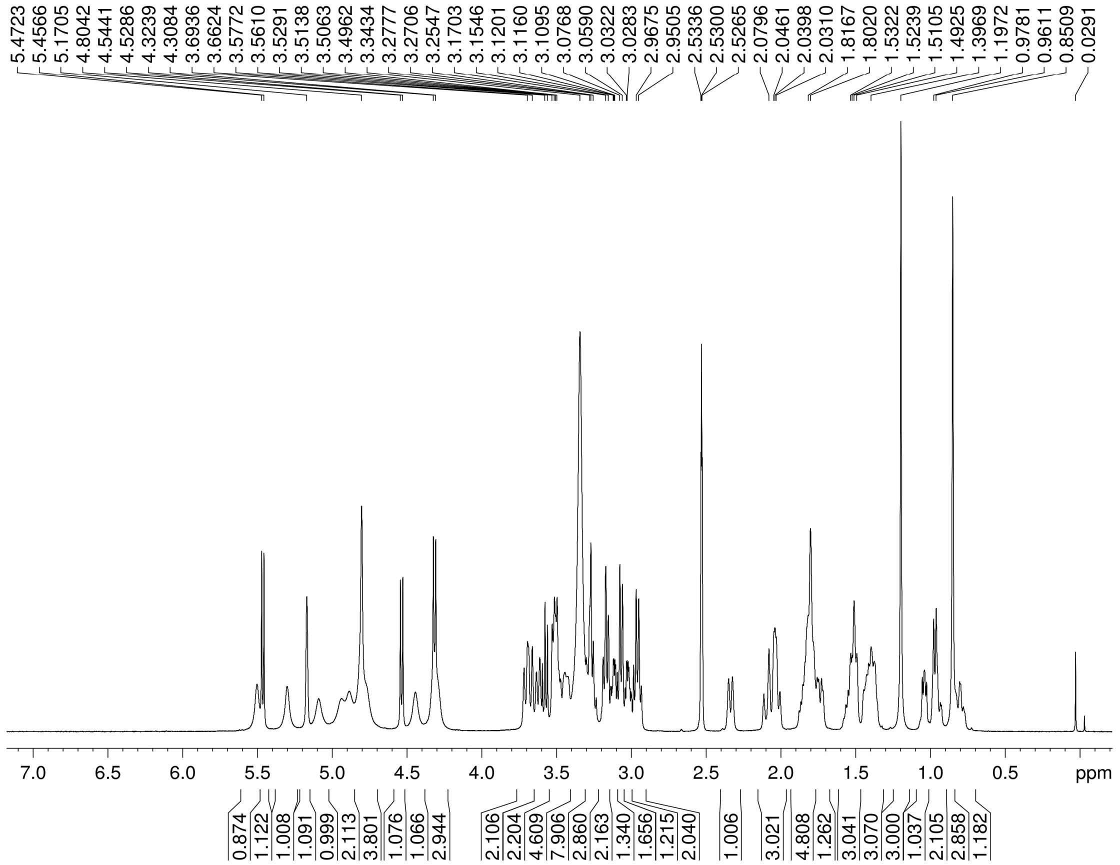

Figure S3. ^{1}H NMR spectrum of compound **2** in DMSO-d_6 (500 MHz).

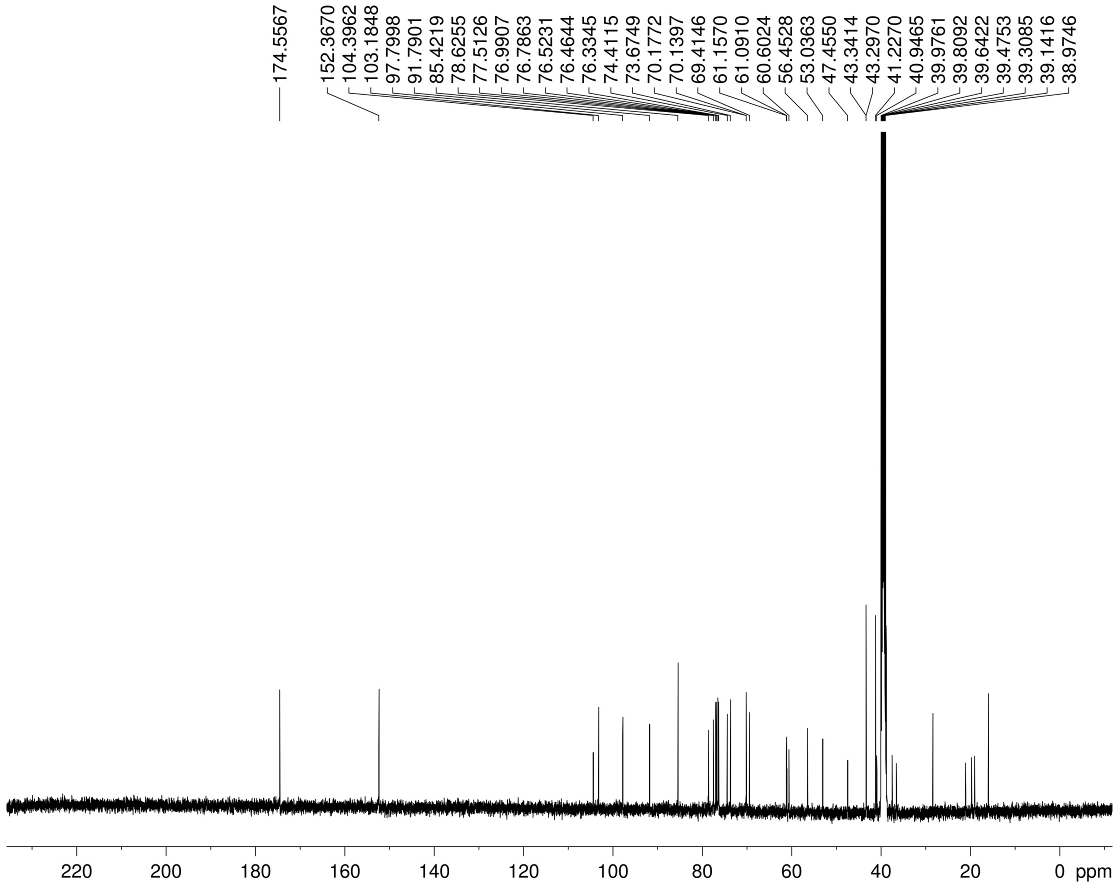

Figure S4. ^{13}C NMR spectrum of compound **2** in DMSO-d_6.

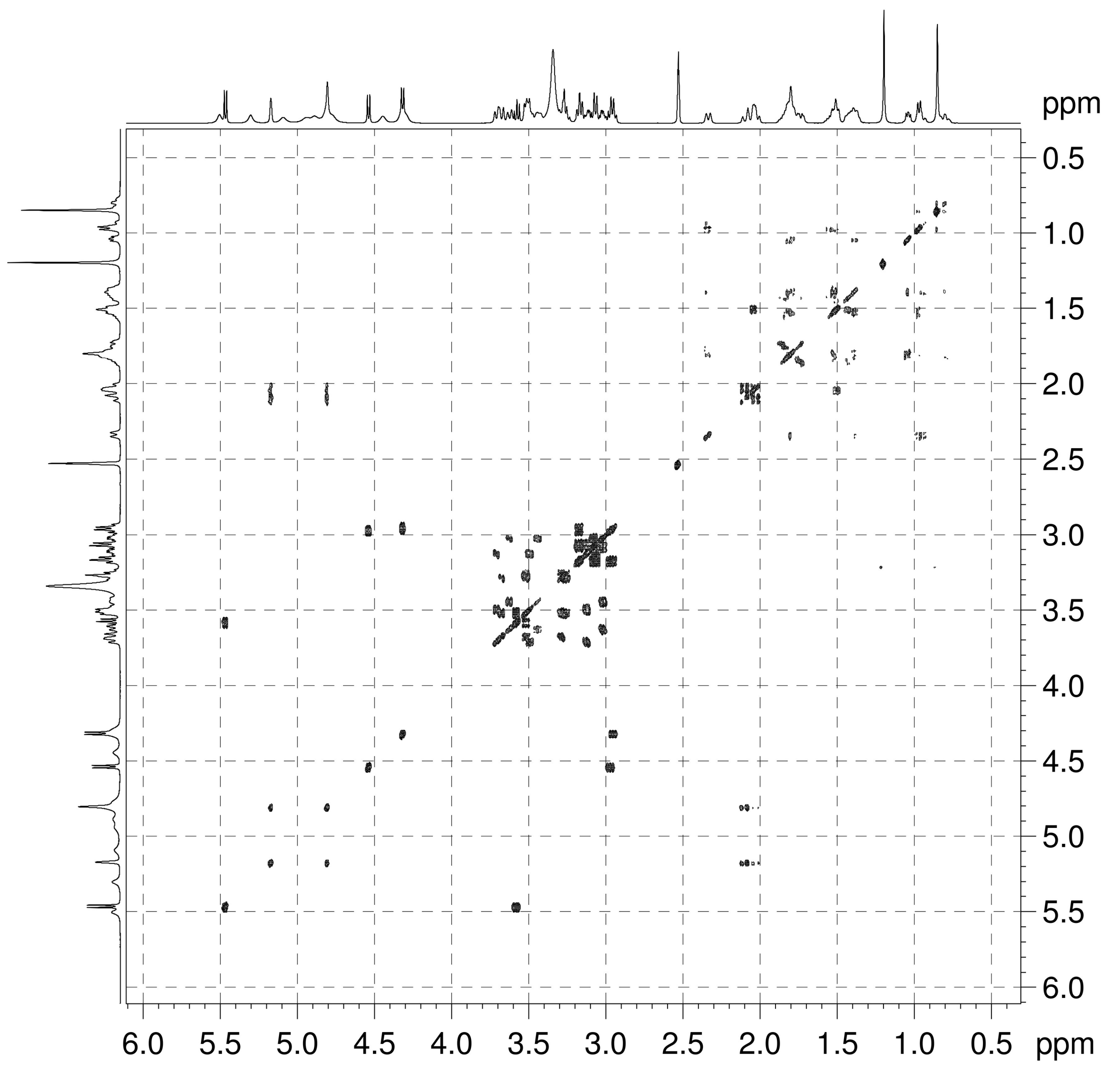

Figure S5. COSY spectrum of compound **2** in DMSO-d_6.

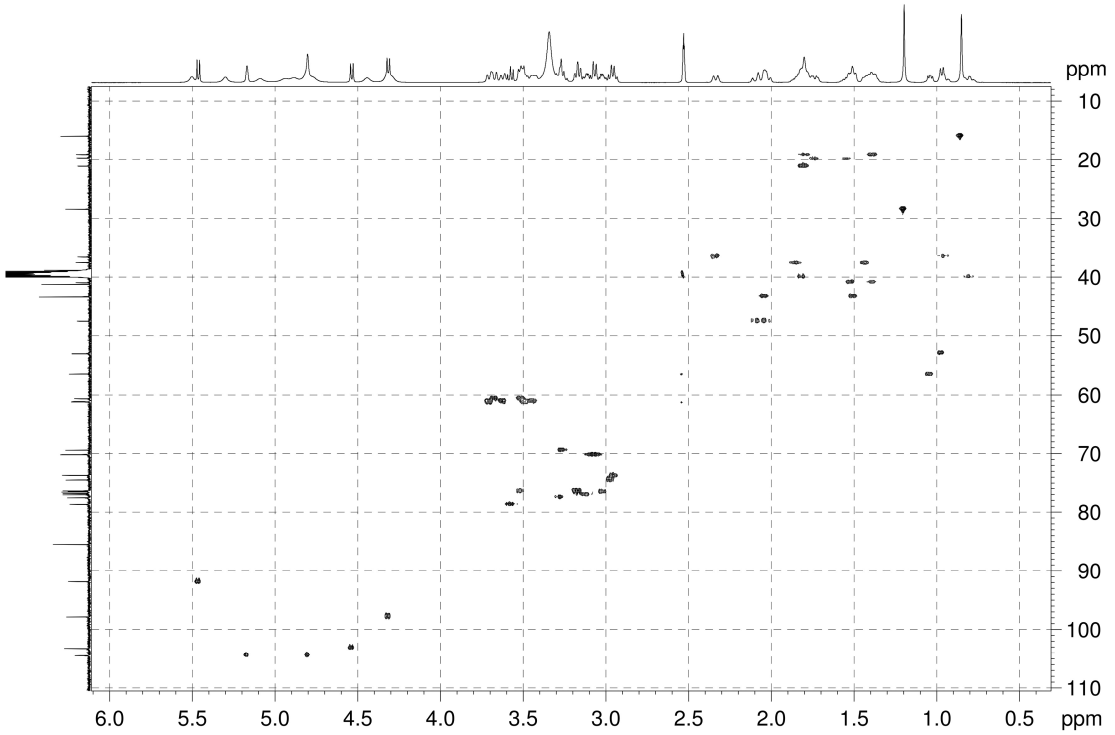

Figure S6. HSQC spectrum of compound **2** in DMSO-d_6.

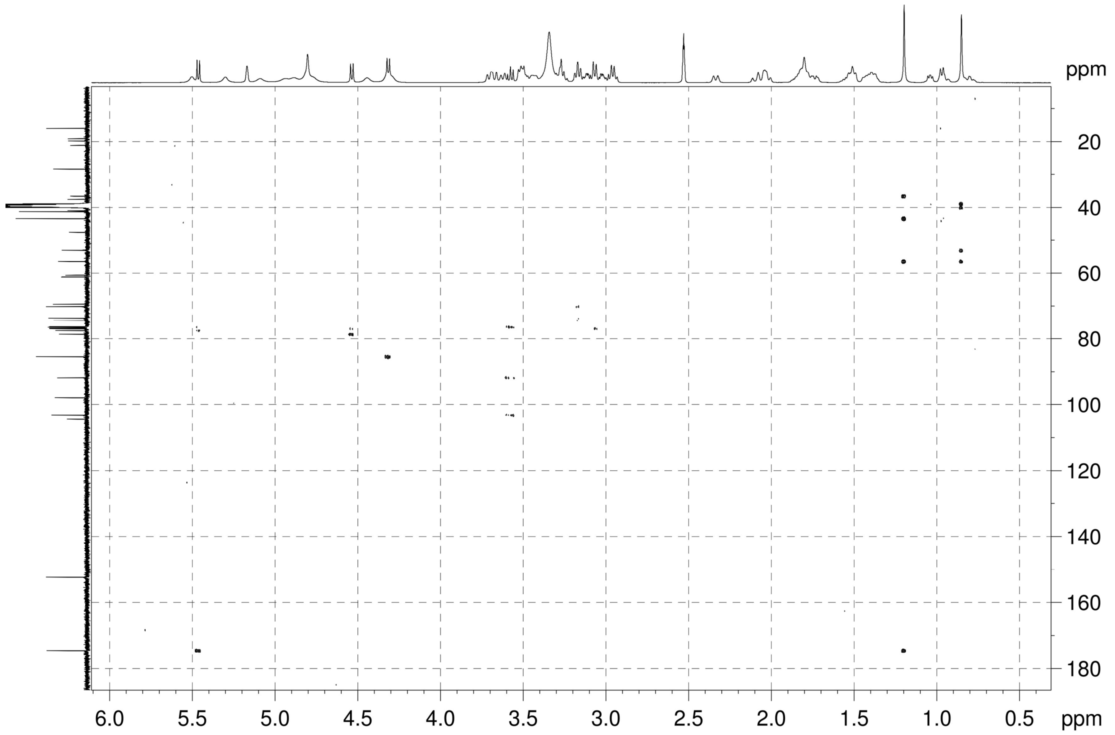

Figure S7. HMBC spectrum of compound **2** in DMSO-d_6.

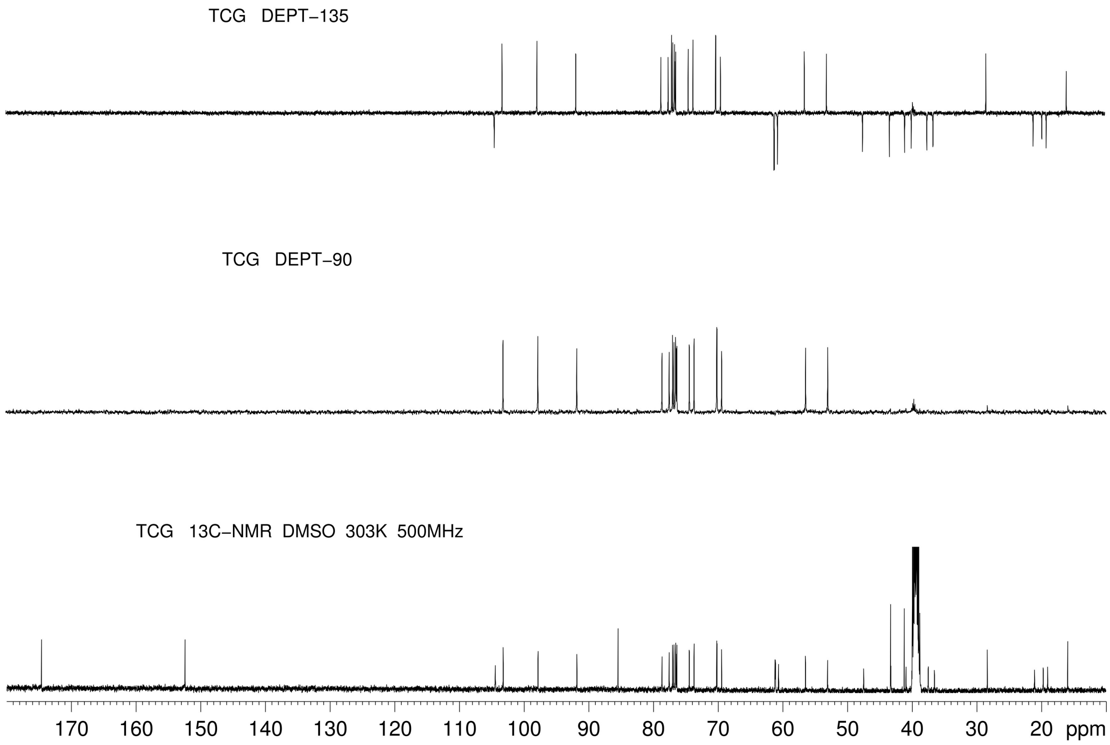

Figure S8. DEPT spectrum of compound **2** in DMSO-d_6.

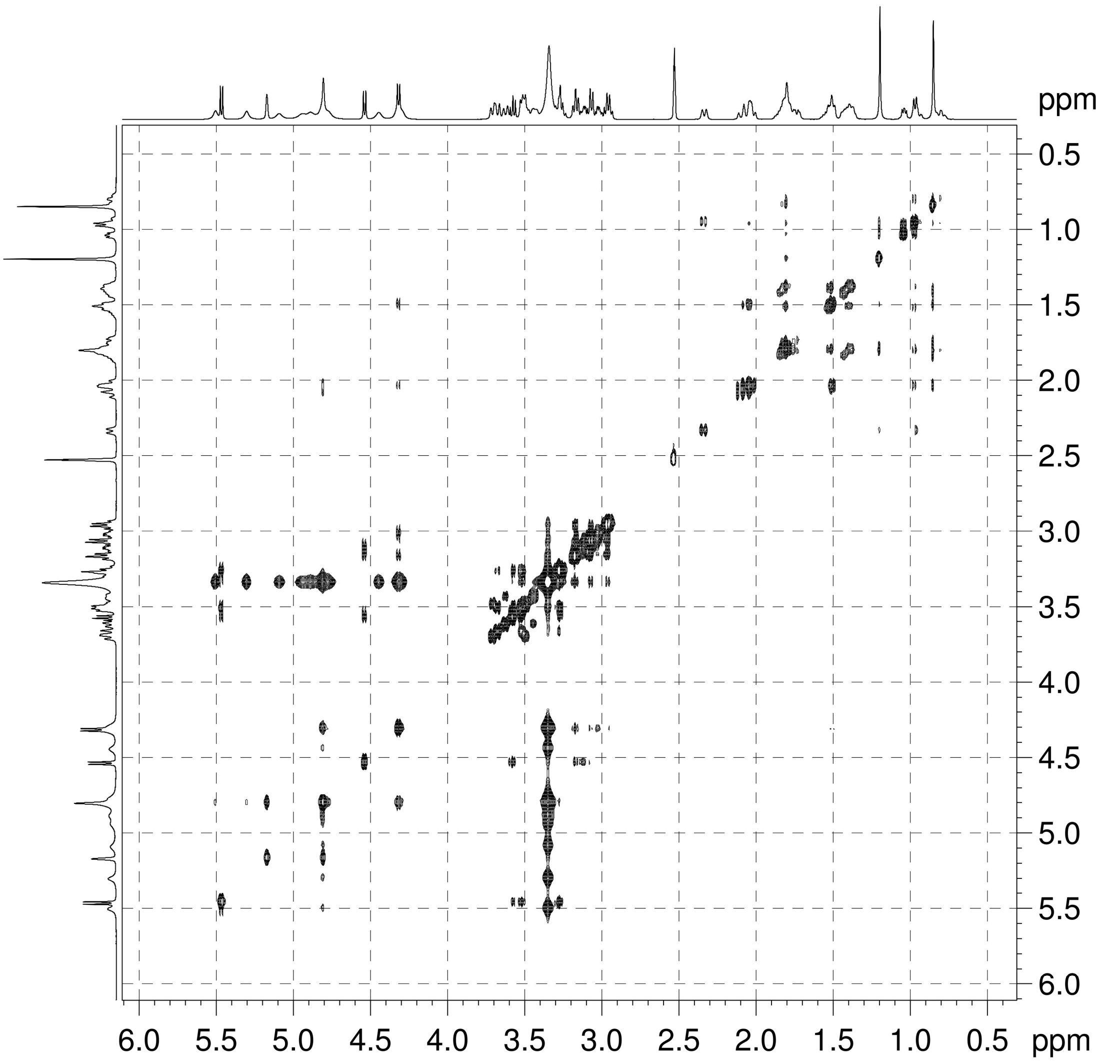

Figure S9. NOESY spectrum of compound **2** in DMSO-d_6.

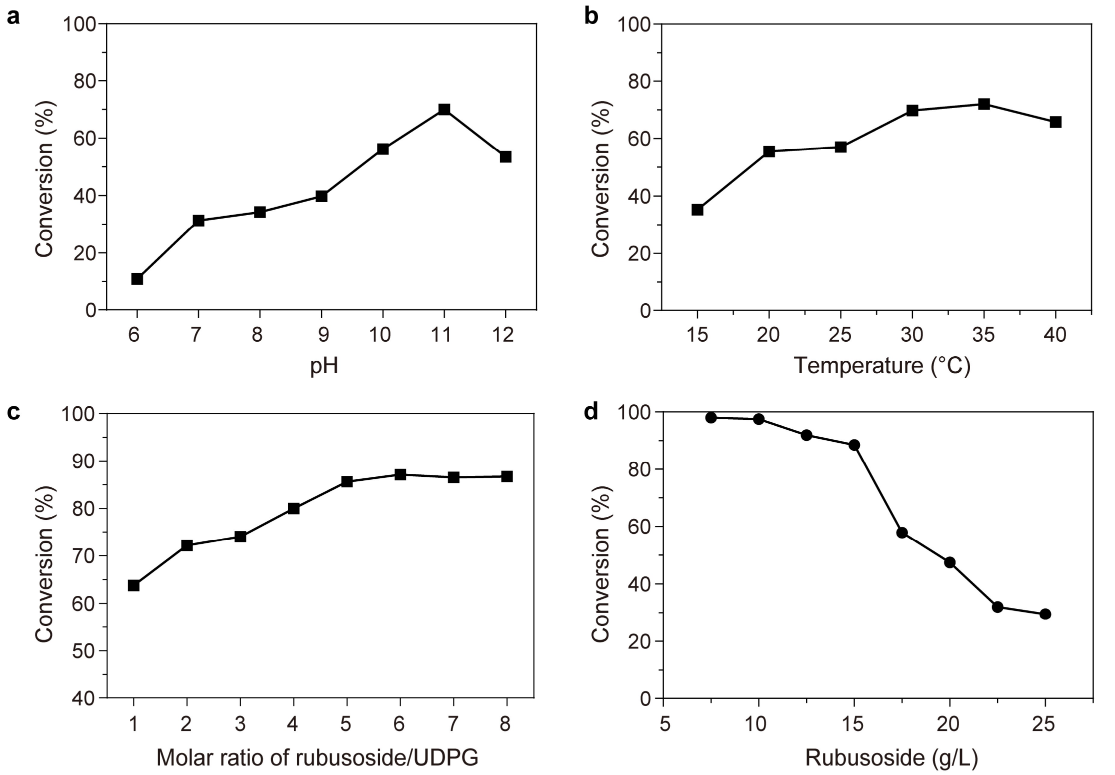

Figure S10. Optimization of reaction conditions for the biosynthesis of rebaudioside KA with crude extract containing OleD.

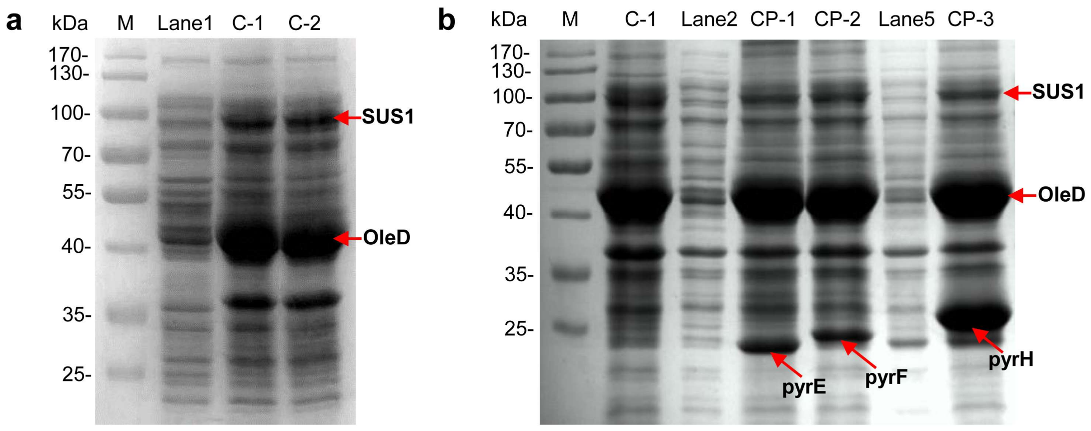

Figure S11. SDS-PAGE of overexpressed proteins in different *E. coli* strains of C-1, C-2, CP-1, CP-2 and CP-3. Lane1, crude extract of *E. coli* BL21(DE3)-pETDuet-1; Lane 2, crude extract of *E. coli* BL21(DE3)-pETDuet-1-pCDFDuet-1; Lane 5, crude extract of *E. coli* BL21(DE3)-pETDuet-1-pACYCDuet-1.

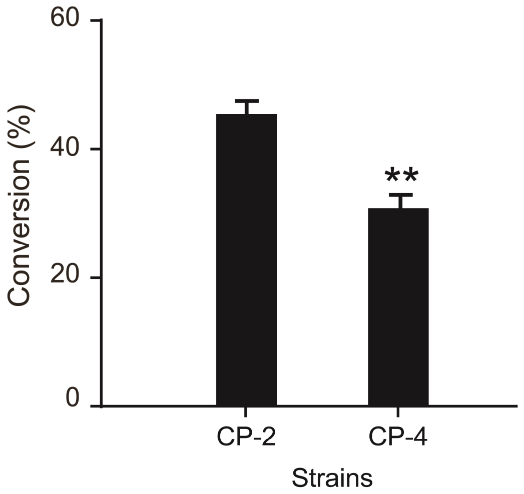

Figure S12. Comparison of rubusoside conversion by the engineered strains. CP-2, pyrF was expressed in *E. coli* strain C-1; CP-4, pyrE, pyrF and pyrH were co-expressed in *E. coli* strain C-1.

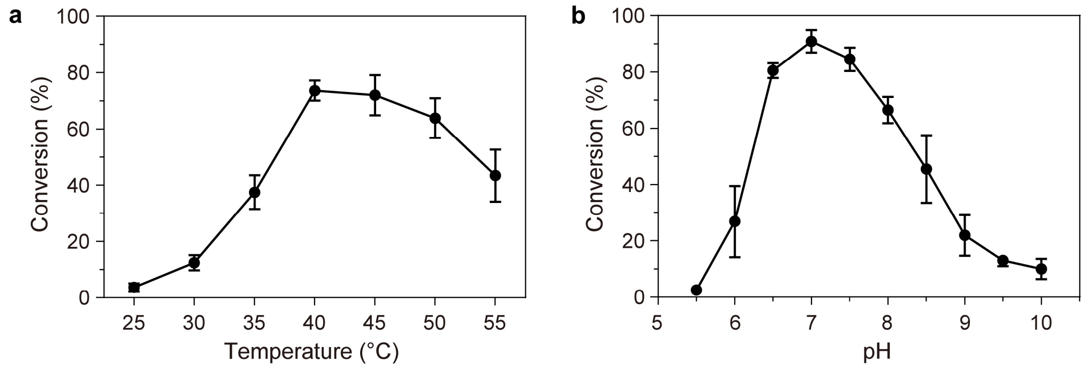

Figure S13. Optimization of reaction conditions for the biosynthesis of rebaudioside KA by engineered *E. coli* strain CPM-2.

Supplementary References

1. Pandey, R. P. *et al.* Assessing acceptor substrate promiscuity of YjiC-mediated glycosylation toward flavonoids. *Carbohydr. Res.* **393,** 26–31 (2014).

2. Williams, G. J., Zhang, C. & Thorson, J. S. Expanding the promiscuity of a natural-product glycosyltransferase by directed evolution. *Nat. Chem. Biol.* **3,**

657–662 (2007).

3. Gantt, R. W., Goff, R. D., Williams, G. J. & Thorson, J. S. Probing the aglycon promiscuity of an engineered glycosyltransferase. *Angew. Chemie Int. Ed.* **47,** 8889–8892 (2008).
4. Losey, H. C. *et al.* Tandem action of glycosyltransferases in the maturation of vancomycin and teicoplanin aglycones: Novel glycopeptides. *Biochemistry* **40,** 4745–4755 (2001).
5. Truman, A. W. *et al.* Chimeric glycosyltransferases for the generation of hybrid glycopeptides. *Chem. Biol.* **16,** 676–685 (2009).
6. Datsenko, K. A. & Wanner, B. L. One-step inactivation of chromosomal genes in *Escherichia coli* K-12 using PCR products. *Proc. Natl. Acad. Sci. U. S. A.* **97,** 6640–6645 (2000).
7. Cherepanov, P. P. & Wackernagel, W. Gene disruption in *Escherichia coli*: Tc^R and Km^R cassettes with the option of Flp-catalyzed excision of the antibiotic-resistance determinant. *Gene* **158,** 9–14 (1995).
